# Zidovudine/Lamivudine for HIV-1 Infection Contributes to Limb Fat Loss

**DOI:** 10.1371/journal.pone.0005647

**Published:** 2009-05-21

**Authors:** Marit G. A. van Vonderen, Michiel A. van Agtmael, Elly A. M. Hassink, Ana Milinkovic, Kees Brinkman, Suzanne E. Geerlings, Matti Ristola, Arne van Eeden, Sven A. Danner, Peter Reiss, for the MEDICLAS study group

**Affiliations:** 1 Department of Internal Medicine, VU University Medical Center, Amsterdam, the Netherlands; 2 International Antiviral Therapy Evaluation Center, Amsterdam, The Netherlands; 3 Hospital Clinic, Barcelona, Spain; 4 Onze Lieve Vrouwe Gasthuis, Amsterdam, the Netherlands; 5 Academic Medical Center, Department of Infectious Diseases, Tropical Medicine and AIDS and Center for Infection and Immunity Amsterdam (CINIMA), Amsterdam, The Netherlands; 6 Helsinki University Central Hospital, Helsinki, Finland; 7 Medisch Centrum Jan van Goyen, Amsterdam, the Netherlands; National AIDS Research Institute, India

## Abstract

**Background:**

Lipoatrophy is known to be associated with stavudine as part of the treatment for HIV infection, but it is less clear if this serious side effect is also related to other nucleoside reverse transcriptase inhibitors like zidovudine. We aimed to determine whether zidovudine-sparing first-line antiretroviral therapy would lead to less lipoatrophy and other metabolic changes than zidovudine-containing therapy.

**Methodology/Principal Findings:**

Fifty antiretroviral therapy-naïve HIV-1 infected men with an indication to start antiretroviral therapy were included in a randomized single blinded clinical trial. Randomisation was between zidovudine-containing therapy (zidovudine/lamivudine+lopinavir/ritonavir) and zidovudine-sparing therapy (nevirapine+lopinavir/ritonavir). Main outcome measures were body composition assessed by computed tomography and dual-energy X-ray absorptiometry scan and lipid profile before and after 3, 12, 24 months of antiretroviral therapy. In the zidovudine/lamivudine+lopinavir/ritonavir group, from 3 months onward limb fat decreased progressively by 684±293 grams (estimated mean±standard error of the mean)(p = 0.02) up to 24 months whereas abdominal fat increased, but exclusively in the visceral compartment (+21.9±8.1 cm^2^, p = 0.008)). In contrast, in the nevirapine+lopinavir/ritonavir group, a generalized increase in fat mass was observed. After 24 months no significant differences in high density lipoprotein and total/high density lipoprotein cholesterol ratio were found between both treatment groups, but total and low density lipoprotein cholesterol levels were higher in the nevirapine+lopinavir/ritonavir group (6.1±0.2 versus 5.3±0.2 and 3.6±0.1 versus 2.8±0.1 mmol/l respectively, p<0.05). Virologic response and safety were comparable in both groups.

**Conclusions/Significance:**

Zidovudine/lamivudine+lopinavir/ritonavir, but not nevirapine+lopinavir/ritonavir in antiretroviral therapy-naïve patients, is associated with lipoatrophy and greater relative intraabdominal lipohypertrophy, suggesting that zidovudine/lamivudine contributes to both these features of lipodystrophy. These findings support to no longer consider zidovudine/lamivudine as one of the preferred possible components of first-line antiretroviral therapy where alternative treatments are available.

**Trial Registration:**

ClinicalTrials.gov NCT 00122226

## Introduction

Lipodystrophy is a common and serious problem associated with HIV infection and its treatment [1]. The alterations in body fat, consisting of lipoatrophy, the loss of subcutaneous fat from arms, legs, buttocks and face, and fat accumulation in the intraabdominal region, neck or trunk, are associated with metabolic derangements including hyperlipidemia and insulin resistance, which may contribute to increased cardiovascular risk [1]. Furthermore, they may be stigmatizing and lead to considerable psychological distress [2].

Stavudine, one of the thymidine analogue (TA) nucleoside reverse transcriptase inhibitors (NRTI), has been associated with a substantial risk for lipoatrophy development [3], [4], and its use in antiretroviral-naïve patients is no longer recommended. Less evidence is available concerning the effect on lipoatrophy development of the TA-NRTI zidovudine as part of initial treatment for HIV. One trial showed that the combination of zidovudine and lamivudine (ZDV/3TC) resulted in less limb fat loss than stavudine in combination with didanosine [5], when used as part of combination antiretroviral therapy (cART).

To explore the role of NRTI in more detail, one strategy is to evaluate alterations of body fat distribution and other metabolic markers in NRTI sparing regimens. We therefore performed a randomized trial comparing the effects of a TA-NRTI sparing regimen composed of nevirapine with lopinavir/ritonavir with those of a TA-NRTI containing regimen of ZDV/3TC with lopinavir/ritonavir in prior cART naïve patients. We hypothesized that patients using ZDV/3TC would develop limb fat loss, in contrast to those in the TA-NRTI sparing group. We currently report the results of the planned primary analysis after 24 months of follow-up.

## Methods

The protocol for this trial and supporting CONSORT checklist are available as supporting information; see Checklist S1, Protocol S1 and Protocol Amendment S1, S2, S3, S4, S5.

### Study design and patients

The MEDICLAS (Metabolic Effects of DIfferent CLasses of AntiretroviralS) trial is a multicenter, multinational, single-blinded, randomized trial comparing the TA NRTI-containing regimen of zidovudine/lamivudine (ZDV/3TC, 300/150 mg bd)+lopinavir/ritonavir (LPV/r, 400/100 mg capsules bd) with the TA NRTI-sparing regimen of nevirapine (NVP, 200 mg bd, following a 2-week 200 mg od lead-in)+LPV/r (533/133 mg bd). To compensate for any increased LPV/r clearance resulting from NVP-associated hepatic enzyme induction, an increased dose of LPV/r was used in combination with NVP to aim for comparable therapeutic LPV plasma concentrations in both trial arms. Patients were included between February 2003 and June 2005, the complete 24 month follow-up ended in June 2007.

Eligible patients were antiretroviral-naïve HIV-1-infected men, 18–70 years old, with an indication to start cART according to international and national guidelines. Excluded were subjects with extreme obesity (body mass index (BMI)>35 kilogram (kg)/m^2^), diabetes mellitus, a history of hyperlipidemia, or use of lipid lowering drugs, nandrolone or testosterone. Patients were recruited from HIV treatment centers in the Netherlands, Spain, Finland and the United Kingdom. The study was approved by the ethics committees of all participating centers, and each patient provided written informed consent. Primary outcome measures were changes in body composition, and metabolic abnormalities, secondary outcome measures were virologic and immunologic efficacy and overall safety.

### Randomisation

At the central study coördinating center a treatment allocation sequence (1∶1 for ZVD/3TC/LPV/r and NVP/LPV/r) was generated. Patients were allocated by minimization according to their BMI (<25 and >25 kg/m^2^). Two separated minimization schemes were used: one for patients that participated in the clamp substudy (published previously [6]) and one for patients who did not participate in this substudy.

### Assessments

Fat distribution, fasting lipid profile and markers of glucose metabolism were assessed at baseline and 3, 12, and 24 months following start of treatment. These assessments of primary outcome measures were performed by blinded investigators in the Hospital Clinic Barcelona for Spanish participants, and in the VU University Medical Center and the Academic Medical Center in Amsterdam for all other participants. Assessments for routine patient care were performed at the participating hospitals by different physicians who were not blinded to treatment allocation.

### Body composition

We measured BMI in kg/m^2^. Waist-to-hip ratio was calculated from waist circumference, measured midway between the lowest rib margin and the iliac crest, and hip circumference, measured at the widest levels over the greater trochanters. Skinfoldthickness using a skinfold caliper were measured at the level of the m. biceps brachii, m. triceps brachii (both halfway the upper arm), subscapular (just below the inferior angle of the scapula) and supra-iliacal (just above the iliac crest, in the midaxillary line), all on the non-dominant side of the body.

Arm circumference was measured halfway between the acromion and olecranon in the non-dominant arm.

Fat weight was measured using a body impedance analyzer (STA BIA, Akern Bioresearch, Italy), and fat percentage was calculated from fat weight and total weight. These measurements were performed by a single blinded investigator in the Netherlands, and another in Spain.

Limb fat, trunk fat, and lean weight were quantified by DEXA (Hologic QDR-4500W, software version whole body v8.26A:5). A standardized single slice abdominal CT-scan was performed from which the area of visceral (VAT) subcutaneous (SAT), and total adipose tissue (TAT) was determined. CT scans were evaluated at the level of the third (L3, in the Netherlands) or fourth (L4, in Spain) lumbar vertebra. Results are shown for the pooled L3 and L4 scans, as changes in VAT, SAT and TAT over time at levels L3 and L4 were highly correlated (in a subset of patients in whom CT scans were performed at both levels at consecutive visits), and results of the analyses after exclusion of L4 scans were comparable to those of all scans combined.

At all visits lipodystrophy scores were calculated according to the complete model of the international lipodystrophy case definition [7].

### Lipid profile and markers of glucose metabolism

Fasting lipid levels included total, high-density (HDL) and low-density lipoprotein (LDL) cholesterol and triglycerides. LDL levels were calculated with the Friedewald formula, if triglyceride values were <4.52 mmol/l. Insulin sensitivity was estimated by the homeostasis model assessment of insulin resistance (HOMA), calculated as (fasting insulin (µU/ml)×fasting glucose (mmol/l))/22.5.

### Virology, immunology and safety

Plasma HIV-1 RNA measurements (lower limit of quantification 50 copies/mL), CD4 cells, routine hematology and chemistry were monitored at baseline, after 1 and 3 months of treatment, and every three months thereafter. The Adult AIDS Clinical Trial Group (ACTG) table for grading severity of adverse experiences was used for reporting of clinical and laboratory adverse events [8].

LPV concentration was analysed centrally by validated HPLC using diode array detection: coefficient of variation 1.6% at 1.98 mg/L and 1.7% at 7.96 mg/L; detection limit 0.008 mg/L. LPV concentration ratios were calculated by dividing the measured concentration by the expected concentration from a reference curve [9].

### Statistical analysis

Analyses were by modified intention-to-treat (intention to treat-exposed), including all randomized patients who received at least one dose of allocated treatment. Within group changes, between-group differences in overall course and between-group differences at study visits were analyzed using a linear model that takes repeated measures into account (Proc Mixed in SAS), with correction for differences in baseline values. The most appropriate covariate structure was selected based on the likelihood ratio test using a restricted maximum likelihood model for estimations. Data are presented as estimated means±standard error of the mean. Data that were not analyzed longitudinally were compared between groups using Mann Whitney U tests and Chi square/Fisher exact tests (where applicable). Measures of body composition were correlated using Pearson correlation. Alpha<0.05 was considered statistically significant. SAS version 9.1 (SAS Institute, Cary, North Carolina, USA)(for the longitudinal analysis) and SPSS statistical software version 15.0 (SPSS Inc, Chicago, IL) were used for the analyses.

## Results

### Patient characteristics

**Figure 1:**
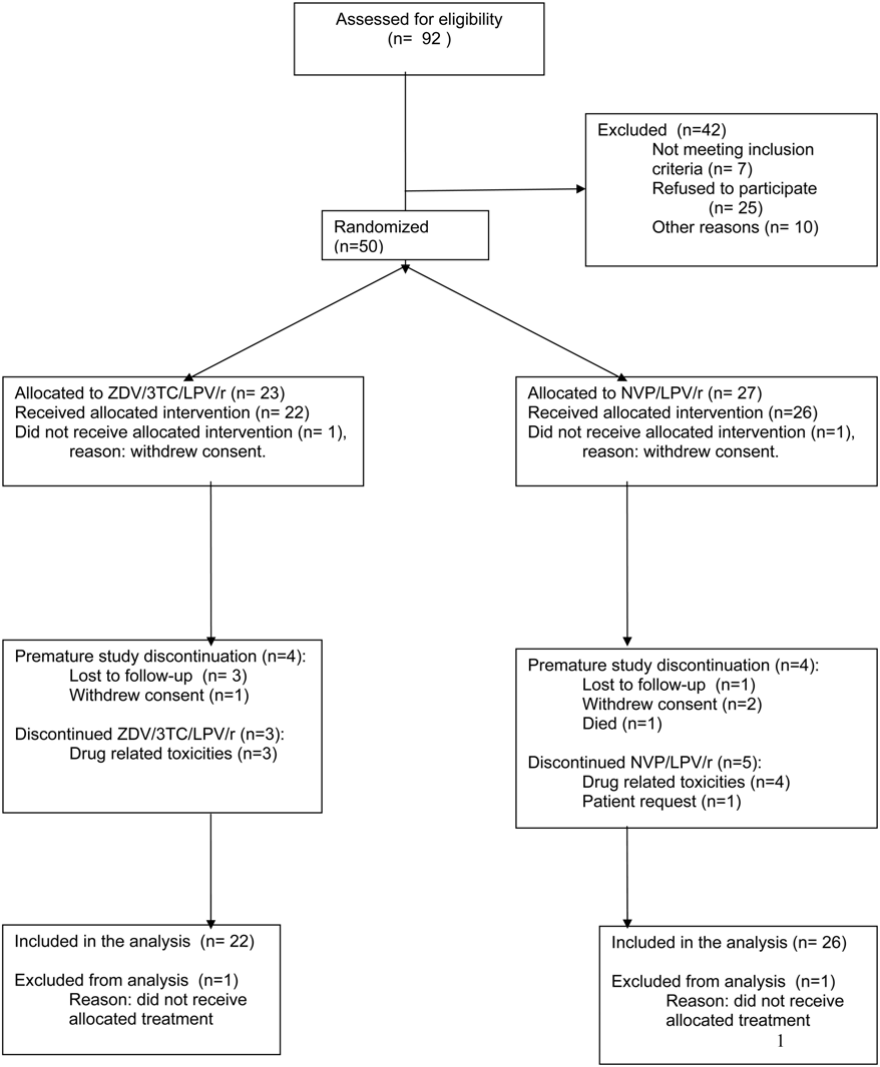
Patient disposition. ZDV/3TC/LPV/r zidovudine/lamivudine/lopinavir/ritonavir. NVP/LPV/r nevirapine/lopinavir/ritonavir.

Fifty patients were included between February 2003 and June 2005; 23 were randomly assigned to ZDV/3TC/LPV/r and 27 to NVP/LPV/r. Two patients, one in each group, did not start allocated therapy and were not included in the analysis (Figure 1). Eight patients, 4 in each group, whose data were included in the intent-to-treat analysis, prematurely discontinued the study (4 were lost to follow-up, 3 withdrew consent and one patient died at home, presumably due to an acute myocardial infarction). The groups had comparable baseline demographic and HIV disease characteristics (Table 1).

**Table 1:** Baseline characteristics of the study population.

	ZDV/3TC/LPV/r (n = 22)	NVP/LPV/r (n = 26)
Age (years)	38 (35–43)	43 (35–52)
Ethnic group		
Black	1 (4.5%)	2 (7.7%)
Caucasian	19 (86.4%)	20 (76.9%)
Hispanic	0 (0%)	2 (7.7%)
Other	2 (9.1%)	2 (7.7%)
Mode of infection		
Heterosexual	2 (9.1%)	3 (11.5%)
Heterosexual/MSM	0 (0%)	1 (3.8%)
MSM	20 (90.9%)	21 (80.8%)
Unknown	0 (0%)	1 (3.8%)
CDC category		
A	14 (63.6%)	16 (61.5%)
B	3 (13.6%)	6 (23.1%)
C	5 (22.7%)	4 (15.4%)
CD4 cell count (10^6^/l)	225 (170–280)	200 (120–360)
HIV-RNA (log 10 copies/ml)	5 (5–5)	5 (5–5)

Shown are median (interquartile range) or number (percentage).

ZDV/3TC/LPV/r zidovudine/lamivudine/lopinavir/ritonavir.

NVP/LPV/r nevirapine/lopinavir/ritonavir.

MSM men who have sex with men.

CDC Centers for Disease Control.

Antiretroviral therapy was modified in eight patients. In the ZDV/3TC/LPV/r group, one patient switched from ZDV to tenofovir after 3 months for anemia, one patient switched to tenofovir/lamivudine/efavirenz after 4 months for anemia and hypercholesterolemia and one patient switched from LPV/r to NVP for hypercholesterolemia after 17 months. In the NVP/LPV/r group, two patients switched to ZDV/3TC/NVP, one after 3 months because of diarrhea and one after 24 months at his own request. In three other patients NVP was replaced by efavirenz because of hepatotoxicity and/or rash after 1, 2 and 5 months, respectively.

### Body composition (Figure 2, Table 2)

Body composition at baseline was comparable between groups.

**Figure 2:**
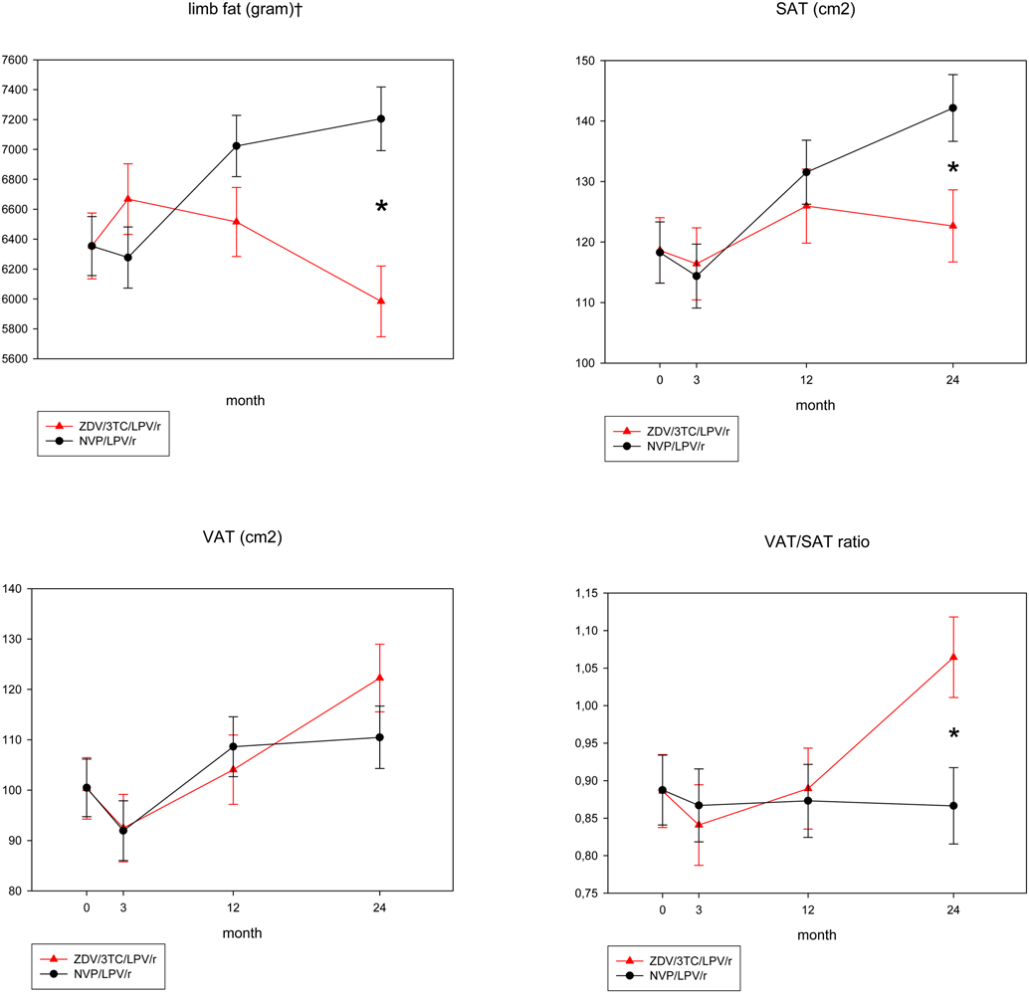
Body composition by Dexa and CT. Shown are estimated means±standard error of the mean (mixed model repeated measures analysis with correction for differences in baseline values). ZDV/3TC/LPV/r zidovudine/lamivudine/lopinavir/ritonavir. NVP/LPV/r nevirapine/lopinavir/ritonavir. *p<0.05 between groups at visit. ‡p<0.05 overall difference between groups.

**Table 2:** Body composition, lipid profile, insulin sensitivity, virology/immunology and lopinavir levels.

	ZDV/3TC/LPV/r				NVP/LPV/r			
	Mo 0	Mo 3	Mo 12	Mo 24	Mo 0	Mo 3	Mo 12	Mo 24
CT/Dexa								
Total arm fat (gram)†	1756±82	1909±88*	1715±85	1680±88* $	1756±74	1590±77	1811±77	1918±79$
Total leg fat (gram)†	4590±160	4750±172	4769±165	4295±172* $	4588±144	4677±149	5203±149	5275±155# $
Trunk fat (gram)	7522±255	7527±274	8237±267	8271±274# $	7492±229	6988±238	8245±238	8438±247# $
Total fat (gram)†	13877±425	14194±456	14751±446	14254±457*	13847±381	13265±396	15269±396	15643±412# $
Lean body mass (gram)	53369±421	53179±440	53842±424	53897±430	53404±431	54431±453	54285±453	53943±465
TAT (cm^2^)	218.7±9.8	208.6±10.8	230.2±11.0	244.7±10.8# $	218.6±9.2	206.3±9.5	240.3±9.5	252.4±9.9# $
Anthropometry								
Weight (kg)	74.6±0.7	74.7±0.7	75.9±0.7	75.6±0.7	74.6±0.6	74.9±0.6	76.9±0.6	77.2±0.7# $
Skinfold subscapular (mm)	20.2±1.1	21.6±1.2	21.0±1.2	20.3±1.2	20.2±1.0	20.9±1.1	22.7±1.1	20.8±1.1
Waist circumference (cm)	87.7±0.7	88.0±0.7	89.6±0.7	89.6±0.7#	87.7±0.6	88.0±0.6	90.4±0.6	90.2±0.7# $
Hip circumference (cm)†	95.9±0.5	95.7±0.5	96.5±0.5*	96.1±0.5*	95.9±0.5	96.2±0.5	98.0±0.5	97.8±0.5# $
Arm circumference (cm)	29.1±0.3	29.1±0.3	29.5±0.3	29.3±0.3	29.0±0.3	29.2±0.3	30.0±0.3	30.0±0.3#
BIA fat percentage (%)	18.6±0.6	18.4±0.6	18.4±0.6	18.1±0.6	18.6±0.5	18.2±0.6	19.7±0.6	19.7±0.6$
Lipid profile (fasting)								
Total cholesterol (mmol/l)†	4.4±0.2	5.3±0.2*	5.4±0.2	5.3±0.2* #	4.5±0.2	5.8±0.2	5.9±0.2	6.1±0.2#
HDL cholesterol (mmol/l)	1.1±0.05	1.2±0.05	1.4±0.05	1.4±0.05# $	1.1±0.05	1.4±0.05	1.4±0.05	1.4±0.05#
LDL cholesterol (mmol/l)	2.7±0.1	2.9±0.1	2.8±0.1	2.8±0.1	2.7±0.1	3.2±0.1	3.1±0.1	3.6±0.1# $
Total/HDL cholesterol	4.3±0.2	4.5±0.2	4.3±0.2	4.2±0.2	4.3±0.2	4.5±0.2	4.7±0.2	4.4±0.2
Triglycerides (mmol/l)	1.4±0.3	2.8±0.4	3.2±0.4	2.8±0.4#	1.4±0.3	2.7±0.3	3.2±0.3	2.4±0.3#
Apolipoprotein A (g/l)	1.0±0.04	1.2±0.04	1.2±0.04	1.4±0.05# $	1.1±0.04	1.2±0.04	1.3±0.04	1.4±0.04# $
Apolipoprotein B (g/l)	0.9±0.04	1.0±0.05	1.0±0.05*	1.1±0.05#	0.9±0.04	1.1±0.05	1.2±0.04	1.2±0.05#
Insulin sensitivity (fasting)								
Glucose	5.1±0.1	4.9±0.1	5.0±0.1	4.9±0.1	5.0±0.1	5.0±0.1	5.1±0.1	5.1±0.1
Insulin	57±6.8	52±7.3	60±7.4	54±7.5	61±6.3	45±7.0	51±6.8	71±7.0
HOMA	1.9±0.2	1.6±0.2	1.8±0.2	1.7±0.2	1.9±0.2	1.5±0.2	1.7±0.2	2.3±0.2
Virology/immunology								
CD4 cell count (×10^6^/l)	220 (170–264)	344 (250–440)	428 (360–557)	502 (385–685)	200 (120–360)	315 (210–520)	370 (284–606)	485 (340–625)
HIV-RNA (log 10 copies/ml)	5.1 (5.0–5.3)	2.1 (1.7–2.5)	1.7 (1.7–1.7)	1.7 (1.7–1.7)	4.9 (4.6–5.3)	1.7 (1.7–2.3)	1.7 (1.7–1.7)	1.7 (1.7–1.7)
Lopinavir levels								
Lopinavir concentration (mg/l)		5.2 (3.1–6.9)	5.2 (3.7–6.6)	5.5 (3.8–6.9)		6.1 (3.7–6.9)	6.3 (5.3–6.9)	8.0 (4.0–9.3)
Lopinavir concentration ratio		1.2 (0.8–1.8)	1.2 (0.8–1.5)	1.0 (0.8–1.6)		1.2 (1.0–1.4)	1.4 (1.0–1.6)	1.6 (1.1–2.1)

Shown are estimated means±standard error of the mean (for variables included in the longitudinal analysis) or median (interquartile range) (for other variables).

ZDV/3TC/LPV/r zidovudine/lamivudine/lopinavir/ritonavir.

NVP/LPV/r nevirapine/lopinavir/ritonavir.

VAT visceral adipose tissue. SAT subcutaneous adipose tissue. TAT total adipose tissue. BIA body impedance analysis. HDL high density lipoprotein. LDL low density lipoprotein. HOMA homeostasis model assessment of insulin resistance.

* p<0.05 between groups at each timepoint.

† p<0.05 overall comparison between groups over time.

# p<0.05 within group 0–24 months.

$ p<0.05 within group 3–24 months.

Body weight remained stable during 24 months in the ZDV/3TC/LPV/r group, whereas it increased in the NVP/LPV/r group. Lean weight remained stable in both groups. The changes in fat mass showed significantly different patterns between the groups. Limb fat remained stable during the first three months in the ZDV/3TC/LPV/r group but progressively declined thereafter, with a total loss of 684±293 grams from 3 to 24 months (p = 0.02). Total fat remained unchanged in this group, resulting in a significant decrease of the limb to total fat ratio (0.47±0.008 to 0.44±0.008; p = 0.0015). In contrast, in the NVP/LPV/r group, limb fat and total fat both increased, with no change in the limb to total fat ratio. After 24 months, limb fat in the ZDV/3TC/LPV/r group was 1223±318 gram lower than in the NVP/LPV/r group (p = 0.0002).

Trunk fat and TAT increased in both groups during follow-up. In the ZDV/3TC/LPV/r group VAT increased (+21.9±8.1 cm^2^ over 24 months (p = 0.008)), while SAT remained unchanged. In contrast, in the NVP/LPV/r group, SAT and VAT both increased after the first 3 months. As a result, the ratio of VAT to SAT increased significantly in the ZDV/3TC/LPV/r group, but remained unchanged in the NVP/LPV/r group, resulting in a significant difference between groups after 24 months.

Of the 40 patients with complete lipodystrophy scores at 24 months, 3/18 (16.7%) in the ZDV/3TC/LPV/r group and 0/22 (0%) in the NVP/LPV/r group had lipodystrophy according to the international case definition (Fisher exact p = 0.083).

**Figure 3:**
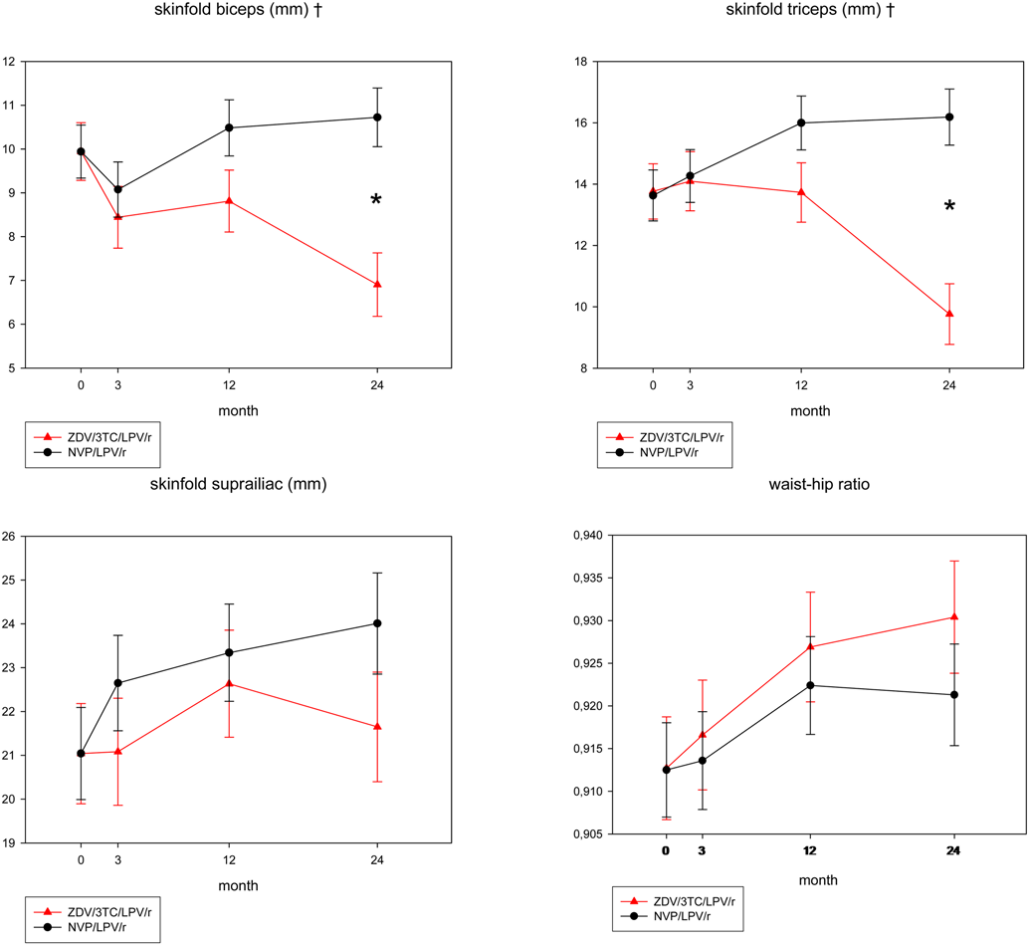
Body composition by anthropometry. Shown are estimated means±standard error of the mean (mixed model repeated measures analysis with correction for differences in baseline values). ZDV/3TC/LPV/r zidovudine/lamivudine/lopinavir/ritonavir. NVP/LPV/r nevirapine/lopinavir/ritonavir. *p<0.05 between groups at visit. ‡p<0.05 overall difference between groups.

Anthropometry and BIA findings were consistent with CT and DEXA results (Figure 3, Table 2). Biceps skinfold thickness decreased by 3.0±0.9 mm (p = 0.0008) and triceps by 4.0±1.2 mm (p = 0.0007) in the ZDV/3TC/LPV/r group, but remained stable (biceps) or increased (triceps) in the NVP/LPV/r group. After 24 months, patients in the ZDV/3TC/LPV/r group had 3.8±1.0 mm (p = 0.0002) lower biceps and 6.4±1.3 mm (p<0.0001) lower triceps skinfold thickness compared to those in the NVP/LPV/r group. Arm circumference did not change in the ZDV/3TC/LPV/r group but increased in the NVP/LPV/r group. Changes in biceps and triceps skinfolds and arm circumference were significantly correlated with changes in limb fat (correlation coefficients 0.21, 0.39 and 0.48, respectively, p = 0.007, 0.007 and <0.0001). Hip circumference increased by 1.9±0.6 cm (p = 0.0019) in the NVP/LPV/r group, but remained stable in the ZDV/3TC/LPV/r group, whereas waist circumference increased in both groups (+2.0±0.9 cm (p = 0.03) and +2.6±0.8 cm (p = 0.002) for the ZDV/3TC/LPV/r and NVP/LPV/r groups respectively). This resulted in a significant increase in the waist-to-hip ratio in the ZDV/3TC/LPV/r group, and no change in the NVP/LPV/r group. Changes in waist circumference were correlated with changes in TAT and VAT (correlation coefficients 0.38 and 0.60, p<0.0001). The suprailiac skinfold remained unchanged in the ZDV/3TC/LPV/r group, but increased in the NVP/LPV/r group (+3.0±1.3 cm, p = 0.03), and changes in suprailiac skinfold correlated with changes in SAT (correlation coefficient 0.50, p<0.001).

### Lipid profile (Table 2)

Total cholesterol increased significantly in both groups (+23.2±5.8% in the ZDV/3TC/LPV/r group compared to +36.5±5.2% in the NVP/LPV/r group; difference between groups 13.3±7.8%, p = 0.09). HDL cholesterol increased significantly by 32.8±7.4% in the ZDV/3TC/LPV/r group and by 38.8±6.6% in the NVP/LPV/r group (difference between groups 6.0±9.9%, p = 0.5). The total over HDL cholesterol ratio did not change significantly over time in either of the groups. Whereas LDL cholesterol did not increase in the ZDV/3TC/LPV/r group (+11.5±7.4%, p = 0.13), a significant rise by 31.1±6.4% was observed in the NVP/LPV/r group, difference between groups 19.6±9.8%, p = 0.05). Triglycerides increased in both groups (+108±30% and +81±27% for the ZDV/3TC/LPV/r and NVP/LPV/r group, respectively), with no significant difference between groups.

### Markers of glucose metabolism (Table 2)

There were no significant overall changes in fasting glucose, insulin and HOMA in either of the treatment groups, with no overall difference between groups.

### Virologic and immunologic parameters and lopinavir concentrations (Table 2)

Patients in both groups had similar immunologic and virologic responses to cART. The median CD4 cell increase over 24 months was 280 (205–455) and 308 (191–410)×10^6^ cells/l in the ZDV/3TC/LPV/r and NVP/LPV/r group, respectively. At 24 months, 17/22 (77%) of the patients in the ZDV/3TC/LPV/r group and 21/26 (80%) in the NVP/LPV/r group had a plasma HIV-RNA below 50 copies/ml. After 24 months there was a trend for the LPV concentration to be greater in the NVP/LPV/r group (8.0 (4.0–9.3) mg/l compared with 5.5 (3.8–6.9) mg/l for the ZDV/3TC/LPV/r group (p = 0.065).

### Adverse events

The proportion of patients who developed grade 3 and 4 adverse events was similar in both groups (10/22 (45.8%) in the ZDV/3TC/LPV/r group and 14/26 (54.2%) in the NVP/LPV/r group, p = 0.77). The most commonly observed adverse events were hyperlipidemia (4/22 (18%) in the ZDV/3TC/LPV/r group and 6/26 (23%) in the NVP/LPV/r group) and gastrointestinal complaints (4/22 (18%) in the ZDV/3TC/LPV/r group and 5/26 (19%) in the NVP/LPV/r group).

## Discussion

The main finding from this trial is that ZDV/3TC contributed to the development of limb fat atrophy in HIV-1-infected men not previously exposed to cART. Whereas limb fat after 24 months had increased by 851±255 grams in patients allocated to NVP/LPV/r, a progressive loss of limb fat commencing after 3 months and reaching a decline of 684±292 grams by 24 months was observed in those randomized to ZDV/3TC/LPV/r. Moreover, while patients randomized to NVP/LPV/r demonstrated a generalized increase in fat mass, including both subcutaneous and intraabdominal compartments, patients assigned to ZDV/3TC/LPV/r experienced selective gain of visceral adipose tissue without any change in the subcutaneous abdominal compartment, which is characteristic for HIV-associated lipodystrophy. Consistent with this observation, lipodystrophy according to the International Case Definition was present in 3/18 patients allocated to ZDV/3TC/LPV/r after 24 months follow-up, but in none of the 22 evaluable patients allocated to NVP/LPV/r. Although neither designed nor sufficiently powered to allow a formal comparison of efficacy, our study demonstrated excellent and comparable virological and immunological efficacy for both regimens, consistent with other studies assessing LPV/r in combination with a NNRTI in naïve patients [10]–[12].

The rate of limb fat loss in patients on ZDV/3TC-containing therapy in our study seems to be of a similar magnitude to what has been reported previously from two randomized trials and one observational cohort study in previously treatment-naïve individuals [5], [13], [14]. One should be cautious however in directly comparing the changes in limb fat in those studies with ours, given the differences in both the frequency and timepoints at which DEXA scans were performed in each of the studies. The role of ZDV in lipoatrophy development is also supported by a study in which patients on ZDV-containing regimens replaced ZDV with either tenofovir or abacavir [15], and one in which patients were randomized to either replace ZDV by tenofovir, or to continue their existing ZDV-containing regimen [16].

Whether LPV/r may also have contributed to lipoatrophy, similar to what has been shown for nelfinavir [5], cannot be ruled out given that patients in both groups received LPV/r. However, recent results from a randomized trial in which antiretroviral naive subjects treated with dual NRTI's plus LPV/r exhibited significantly less loss of limb fat than those treated with two NRTI's plus efavirenz would argue against this [17].

Both treatments in our study resulted in similar degrees of intraabdominal fat gain. This may represent an improvement in patients' general health as a result from suppressing HIV, although a contribution from the use of LPV/r cannot be ruled out. Given that all patients received LPV/r and after 24 months there was a trend for patients on ZDV/3TC to have gained more intraabdominal fat, a contribution from these NRTI to this feature of lipodystrophy can also not be discarded. The finding that visceral fat accumulation in patients on ZDV/3TC/LPV/r was observed in the context of stable SAT, whereas in patients on NVP/LPV/r SAT increased in parallel with VAT would seem to support this.

In contrast to the advantages with regard to body composition, the TA sparing regimen of NVP/LPV/r did not have an unequivocal favorable impact on the lipid profile. Compared to patients treated with ZDV/3TC/LPV/r, more patients developed high total and LDL cholesterol levels according to NCEP criteria. However, due to the rise in HDL cholesterol, the total/HDL cholesterol ratio, a powerful predictor of cardiovascular risk [18], did not increase over time. Our findings are consistent with the dyslipidaemia described in patients switching to regimens of LPV/r with efavirenz [19], but contrast with results from another small trial which also included a regimen of NVP/LPV/r in treatment-naive subjects [11]. Adjustment for differences in LPV levels between treatment groups did not change the results of the analysis, suggesting that this is not a likely explanation for the higher total- and LDL-cholesterol levels found in the NVP/LPV/r group. Differences in ritonavir exposure may have influenced the results, but plasma (or intracellular) ritonavir concentrations levels were not measured. HDL levels increased more in the NVP/LPV/r group than in the ZDV/3TC/LPV/r group, but this difference did not reach statistical significance, other than what may have been expected from prior studies [20]. A possible explanation for this is that our sample size was only sufficient to demonstrate a statistically significant difference between groups if this was 33% (or 0.29 mmol/l) or greater.

Several changes in anthropometric measures of body composition showed a good correlation with changes objectively determined by DEXA and CT. This suggests that consecutive measurements of skinfold thicknesses in the upper arm and of the waist-to-hip ratio, may be able to reliably detect the onset of limb fat loss and abdominal fat accumulation. This could potentially be particularly relevant in resource limited settings, where it could guide clinicians in the earlier detection of fat distribution changes and timely modification of cART to try and prevent further deterioration.

As novel antiretroviral drugs are being introduced, alternative TA-sparing strategies become feasible, including regimens employing non-TA NRTI, PI – NNRTI combinations with other PI than LPV/r, and also NRTI sparing regimens incorporating CCR5- and integrase inhibitors. Long term prospective evaluation of such alternative treatment strategies incorporating assessments of body composition and metabolic effects may identify first-line regimens which lack detrimental effects on both body composition and lipid and glucose metabolism.

In conclusion, treatment with ZDV/3TC/LPV/r, in contrast to NVP/LPV/r, during 24 months in cART-naïve men was associated with significant limb fat loss and visceral but not subcutaneous abdominal fat accumulation. This adds to the evidence to remove ZDV/3TC from the list of preferred components of first-line antiretroviral regimens.

## Supporting Information

Checklist S1 CONSORT checklist
(0.11 MB TIF)

Protocol S1 Trial Protocol
(0.10 MB DOC)

Protocol Amendment S1 MEDICLAS study protocol
(0.02 MB DOC)

Protocol Amendment S2 MEDICLAS study protocol
(0.03 MB DOC)

Protocol Amendment S3 MEDICLAS study protocol
(0.03 MB DOC)

Protocol Amendment S4 MEDICLAS study protocol
(0.03 MB DOC)

Protocol Amendment S5 MEDICLAS study protocol
(0.03 MB DOC)
