# Supplementary material for: Zidovudine/Lamivudine for HIV-1 Infection Contributes to Limb Fat Loss
Source: PLoS One. 2009 May 21;4(5):e5647. doi: 10.1371/journal.pone.0005647 (PMC2682584; doi:10.1371/journal.pone.0005647)
Supplement: Protocol S1 — Trial Protocol (0.10 MB DOC) [file pone.0005647.s002.doc]

**Title:**

The MEDICLAS study (**M**etabolic **E**ffects of **Di**fferent **Cl**asses of **A**ntiretroviral**S**)

**Date**: 21-03-02

**Protocol chairs**:

Prof. dr. S.A. Danner, Vrije Universiteit Medisch Centrum, Department of Internal Medicine (Principal Investigator)

Dr. P. Reiss, Academic Medical Centre, Department of Internal Medicine, National AIDS Therapy Evaluation Centre

**Protocol team**:

Drs. M.G.A. van Vonderen, Vrije Universiteit Medisch Centrum, Department of Internal Medicine and Academic Medical Centre, Department of Internal Medicine (Study coordinator)

Dr. S.E. Geerlings, Vrije Universiteit Medisch Centrum, Department of Internal Medicine

Prof. Dr. C.D.A. Stehouwer, Vrije Universiteit Medisch Centrum, Department of Internal Medicine

Prof. Dr. H. P. Sauerwein, Academic Medical Centre, Department of Endocrinology and Metabolism

Drs. M. van der Valk, Academic Medical Centre, Department of Internal Medicine

Dr G.J. Weverling, Academic Medical Centre, Department of Epidemiology

Prof. Dr. C.J.L.M. Meijer, Dr J.J. Oudejans, Vrije Universiteit Medisch Centrum, Department of Pathology

Dr. C. van Kuijk, Academic Medical Centre, Department of Radiology

Prof. Dr. B.L. van Eck-Smit, Academic Medical Centre, Department of Nuclear Medicine

Prof. Dr. R.A. Manoliu, Vrije Universiteit Medisch Centrum, Department of Radiology

Dr. J.C. Roos, Vrije Universiteit Medisch Centrum, Department of Nuclear Medicine

Dr. J.C. Netelenbos, Vrije Universiteit Medisch Centrum, Department of Endocrinology

M. van der Bokhorst, Vrije Universiteit Medisch Centrum, Dietician

**Participating centers**:

Vrije Universiteit Medisch Centrum, Amsterdam (Principal Investigator: Dr. S.E. Geerlings)

Academic Medical Centre, Amsterdam (Principal Investigator: Dr. P. Reiss)

Onze Lieve Vrouwe Gasthuis Amsterdam, location Oosterpark (Principal Investigator: Dr K. Brinkman

Onze Lieve Vrouwe Gasthuis Amsterdam, location Prinsengracht (Principal Investigator: Dr J.H. ten Veen)

Slotervaart Ziekenhuis, Amsterdam (Principal Investigators: Dr J.W. Mulder, Dr P.L.Meenhorst)

Medisch Centrum Jan van Goyen, Amsterdam (Principal Investigator: Dr A. van Eeden)

Kennemer Gasthuis, lokatie Elisabeth Gasthuis, Haarlem (Principal Investigator: Dr. R. ten Kate)

Leids Universitair Medisch Centrum, Leiden (Principal Investigator: Dr F. Kroon)

Academisch Ziekenhuis Dijkzigt, Rotterdam (Principal Investigator: Dr M.E. van der Ende)

Ziekenhuis Leyenburg, Den Haag (Principal Investigator: Dr R. Kauffmann)

**Sponsors**: Abbott, Boehringer

**Table of contents**

1. List of abbreviations and definitions 3
2. Summary 4
3. Background and rationale, including hypotheses 5
4. Study questions 8
5. Methods 9
   1. Subjects 9
   2. Inclusion criteria 9
   3. Exclusion criteria 9
   4. Trial design 9
   5. Randomisation and blinding 9
   6. Intervention/ study arms 10
   7. Usual patient care 10
   8. Description of specific methods 10
   9. Possible side effects and actions to side effects that are

specifically studied in this study 13

1. Data 13
   1. Data recording 13
   2. Statistical analysis 13
   3. Data presentation and publishing 13
2. Ethical considerations 14
   1. General 14
   2. Recruitment, consent and withdrawal 14
   3. Description of burden and possible risk of the study for

subjects enrolled in the study 14

- 1. Subject privacy and data handling 15
  2. Independent physician 15
  3. Unexpected findings 15
  4. Compensation for injury 15

1. Study duration 15
2. References 16
3. Addendum: Flow chart 18

# 1. List of abbreviations and definitions

Abbreviations:

AMC Academic Medical Centre

BIA Bio-electrical Impedance Analysis

CDC Centers for Disease Control

CT Computer Tomography

DEXA Dual Energy X-ray Absorptiometry

ELISA Enzyme Linked Immunosorbent Assay

FFA Free Fatty Acid

HAART Highly Active Antiretroviral Therapy

HIV Human Immunodeficiency Virus

LDL/HDL cholesterol Low/high density lipoprotein cholesterol

MtDNA Mitochondrial DNA

NASBA technique Nucleic Acid Sequence-Based Amplification

nDNA nuclear DNA

NNRTI Non-Nucleoside Reverse Transcriptase Inhibitors

NRTI Nucleoside Reverse Transcriptase Inhibitors

PCR Polymerase Chain Reaction

PI Protease Inhibitors

PBMC’s Peripheral Blood Mononuclear Cells

VUMC Vrije Universiteit Medical Centre

WHO World Health Organisation

Definitions:

Lipodystrophy

Until a clear definition of lipodystrophy is formulated, we will use this term only for the body shape abnormalities (peripheral lipo-atrophy and central lipohypertrophy or a combination of these two) and not for the metabolic disturbances which are found in patients using HAART.

At this moment an objective, validated and readily applicable case definition of HIV lipodystrophy is being formulated by an International Study Group. The model used will include trunk: peripheral fat ratio (DEXA), low leg fat % (DEXA), higher waist-hip ratio, higher total: HDL-cholesterol ratio, higher aniongap, higher visceral: subcutaneous fat ratio (CT) and greater age (1).

**2. Summary**

Treatment of human immunodeficiency virus type 1 (HIV-1) infection has evolved dramatically over the last years. Combination of several antiretroviral agents (highly active antiretroviral therapy (HAART)) leads to profound and sustained suppression of HIV-1 replication. This has resulted in a decline of HIV-related morbidity and mortality. However, the use of these agents is associated with significant middle/long term adverse effects. Some of these effects consist of changes in body fat distribution (peripheral lipo-atrophy and central lipo-hypertrophy, together named lipodystrophy), and changes in carbohydrate and lipid metabolism (insulin resistance and hyperlipidemia). Loss of bone mineral density and premature osteoporosis is another finding that is possibly associated with HAART. Concern is rising that the improvement in life expectancy due to HAART will be (partly) counteracted by an increase in cardiovascular morbidity and mortality as a result of some of the above mentioned metabolic changes. Additionally the changes in body fat distribution significantly affect quality of life for many patients.

The role of the different classes of antiretrovirals has not been completely elucidated. Protease inhibitors and nucleoside reverse transcriptase inhibitors both contribute to different aspects of the syndrome, but the interaction of these drug classes when given in combination therapy seems to greatly increase the risk. Non nucleoside reverse transcriptase inhibitors (NNRTI) might play a protective role in the metabolic changes, given their property to increase HDL-cholesterol levels in contrast to what is seen with protease inhibitors (PI).

Nearly all studies on lipodystrophy are cross-sectional. Prospective data are hardly available and this is also the case for studies about the pathological changes in the most affected tissue: the adipose tissue. Macrovascular measurements such as intima media thickness of the carotid artery and large artery function as determined with distensibility and compliance measurements, which have been shown to be structural and functional markers of the atherosclerotic process in non HIV-infected patients, and microvascular function in relation to insulin resistance have hardly been studied in HIV-infected patients.

We will study changes in body fat distribution, insulin resistance, lipid profiles, bone mineral density, micro-and macrovascular function and pathological changes in adipose tissue in HIV-infected HAART-naïve patients. Patients will be randomised to receive either a PI in combination with a NRTI or a PI in combination with a NNRTI.

The aim of the study is to investigate the occurrence of above mentioned parameters after start of therapy and to compare the degree and timing of occurrence of these parameters between the two groups.

## **3. Background and rationale**

Treatment of human immunodeficiency virus type 1 (HIV-1) infection has evolved dramatically over the last years. Combination of several antiretroviral agents leads to profound and sustained suppression of HIV-1 replication. This has resulted in a decline of HIV-related morbidity and mortality (2,3). However, the use of these agents is associated with significant middle/long term adverse effects. Some of these effects consist of changes in body fat distribution (lipodystrophy) and changes in carbohydrate and lipid metabolism (4).

The changes in body fat distribution are the most visible. These consist of peripheral lipo-atrophy (arms, legs and face) and central lipo-hypertrophy (fat accumulation in the abdomen, dorsocervical fat pad, breast enlargement and sometimes multiple lipomas). Estimates of prevalence of lipodystrophy vary widely up to 83% in cross-sectional studies depending on definition and method of diagnosis (5). Incidence increases with duration of therapy. In one prospective study in patients using PI, the cumulative (self-reported) incidence was 23% after 24 months (6).

Currently, the methods most frequently used for the objective measurement of body fat distribution are DEXA to determine the amount of peripheral adipose tissue (7) and single-slice CT (at the level of the fourth lumbar vertebra) to determine the amount of visceral adipose tissue and subcutaneous adipose tissue (8). Many studies use only self-reported fat wasting or fat accumulation, confirmed with physical examination and bedside tests for body composition (anthropometry (skinfold thickness measurements) and bio-electrical impedance analysis (BIA)). BIA measures total body electrical resistance to calculate lean body mass. However, agreement between these methods and between patient and physician rating is poor and may explain the wide ranges in prevalence of lipodystrophy found in various studies (9). We expect to find a higher incidence of lipodystrophy when using objective measurements of body fat distribution, as has recently been demonstrated to be the case in a pediatric population (10).

The metabolic syndrome associated with HAART consists (among others) of insulin resistance and hyperlipidemia. The hyperinsulinemic euglycemic clamp is currently the best available standard technique to measure insulin resistance (11). Studies using this technique found insulin resistance in healthy subjects following 4 weeks of indinavir therapy (12) and higher insulin resistance in HIV-infected patients with lipodystrophy compared with HIV-infected patients without lipodystrophy and healthy controls (13). Most studies however have used only fasting glucose and insulin levels, sometimes combined with glucose tolerance tests, which provide only imprecise estimates of insulin resistance.

The lipid and lipoprotein profile associated with PI-containing HAART consists of elevated plasma values of total cholesterol, LDL-cholesterol, triglycerides, apolipoprotein B and lipoprotein (a). Prevalences may vary according to used specific antiretroviral compounds and cut-off values used. The prevalence of hyperlipidemia in a cross-sectional study of PI-treated patients was 71 % (82% hypercholesterolemia, 66% hypertriglyceridemia, 46% elevated LDL-cholesterol) (14). In contrast, combination therapy including nevirapine, a NNRTI, was found to be associated with an anti-atherogenic lipid profile with an increase in HDL-cholesterol (15).

In addition to the above mentioned metabolic effects, elevated blood pressure, another risk factor for the development of atherosclerosis, has been described in subjects with lipodystrophy (16).

Recently, a possible association of osteoporosis with HAART use was described. In a cross-sectional study, a decreased whole body, lumbar spine and proximal femur bone mineral density was found in patients using PI containing HAART compared to patients using HAART without PI and HIV negative controls (17). In addition, investigators of another study reported reduced total bone mineral density in 26% of NRTI recipients, 25% of NRTI and PI recipients and 6% of drug-naïve patients (18).

The pathogenesis of lipodystrophy and metabolic changes remains unclear. The different clinical features may possibly be associated with the different drug classes used in HAART. PI use seems to be associated mainly with the metabolic part of the syndrome and with the lipohypertrophy. One of the hypotheses for the mechanism is an interaction of the PI with proteins that regulate lipid metabolism, resulting in hyperlipidemia contributing to central fat deposition and insulin resistance (19). NRTI use on the other hand seems to increase the risk of peripheral fat wasting. NRTI-induced inhibition of the enzyme DNA polymerase gamma, leading to depletion of cellular mtDNA through inhibition of mtDNA synthesis, has been postulated to be a possible underlying mechanism (20). This leads to mitochondrial dysfunction which would be predicted to produce lactic acidosis and cell death through apoptosis.

Recently a method has been described for quantification of mtDNA relative to nDNA in peripheral blood mononuclear cells (PBMC’s), which could possibly become a useful test for indirectly assessing mitochondrial dysfunction (21). Significant reductions in the ratio of mtDNA to nDNA in PBMC’s were found in patients with symptomatic, NRTI-related hyperlactatemia, an effect that resolved after discontinuation of therapy (22). Another study found no evidence for NRTI-associated mtDNA depletion in PBMC’s, but did not relate this to the amount of nDNA (23). In cross-sectional studies, depletion of mtDNA and increasing apoptosis has been demonstrated in subcutaneous fat samples from individuals with lipodystrophy (24,25).

Osteoporosis has also been postulated to result from mitochondrial toxicity (18). The proposed mechanism would be buffering of lactate by hydroxyapatite from the bone. However, cross-sectional studies provide conflicting evidence for this hypothesis (17,18).

The interaction between PIs and NRTIs when taken in combination regimens greatly increases the risk of lipodystrophy, as seen in trials in which a NRTI is added to dual PI regimens (26) and in trials in which dual NRTIs are compared with combinations of PIs and NRTIs (27). The role of NNRTIs in lipodystrophy and metabolic changes is not yet clear, although they are postulated to play a protective role in metabolic changes. Results of switching studies indicate that replacement of a PI by a NNRTI is followed by at least partial reversal of hyperlipidemia and insulin resistance (28) and in one study also of self-reported changes in body shape (29).

The long term consequences of these body shape changes and metabolic abnormalities in HIV-infected patients are still unknown. In non-HIV-infected persons, the constellation of abdominal obesity, hypertension, elevated serum triglycerides and insulin resistance, also named syndrome X, greatly increases the risk of premature mortality due to cardiovascular disease (30). Elevation in plasma levels of LDL-cholesterol is also a known risk factor for cardiovascular disease. Concern is therefore rising that the improvement in life expectancy due to HAART will be (partly) counteracted by an increase in cardiovascular morbidity and mortality as a result of the above mentioned adverse events. There are some case-reports on vascular complications in young patients using HAART (31).

In non-HIV-infected individuals, measurement of carotid intima-media thickness by ultrasonography is used as a marker of atherosclerosis development and a predictor of cardiovascular events (32, 33). In a cross-sectional study in HIV-infected patients 52.7% of PI-users was found to have carotid intima-media thickness > 1 mm compared to 14.9% in HIV-infected patients without PI use and 6.7% in a non-HIV-infected control group (34). Large artery function as determined with distensibility and compliance measurements has not been evaluated in HIV-infected patients.

Microvascular function (capillary recruitment induced by post-occlusive reactive hyperemia and endothelium-dependent vasodilatation in response to iontophoresis of acetylcholine) has been shown to be strongly correlated with insulin sensitivity in normal subjects (35). In the same study an inverse correlation of capillary recruitment with blood pressure was found, leading to the hypothesis that microvascular function may be a common antecedent linking insulin sensitivity to blood pressure. Microvascular function has to our knowledge not been assessed in HIV-infected patients receiving HAART.

We want to study the degree and timing of occurrence of body shape abnormalities, insulin resistance, lipid and lipoprotein abnormalities and bone mineral density changes in patients with HIV before and after starting with HAART. In addition we will study macrovascular morphology and function as an indication of atherosclerosis development and microvascular function because of its known association with insulin resistance and hypertension. Adipose tissue assessment for apoptosis and quantitative mtDNA determination relative to nDNA in PBMC’s and adipose tissue will also be done to gain insight into the underlying pathogenetic mechanism for (part of ) the above mentioned adverse events due to HAART.

Only male subjects will be included because of clear sex differences in lipodystrophy and metabolic disturbances and because of relatively small study size (36,37).

Because of the postulated role of NRTI’s in some of the above mentioned effects, we will compare a NRTI-including regimen with a NRTI-sparing regimen.

We hypothesize that:

1. lipodystrophy and metabolic disturbances will occur in the majority of these patients after starting HAART during the follow-up period of 2 years, and
2. the peripheral lipoatrophy and some of the metabolic disturbances may be less pronounced in the group using the combination of the PI and the NNRTI due to the absence of the interaction between the PI and the NRTI, and
3. micro-and possibly macrovascular function abnormalities will appear in a part of these patients after starting HAART during the follow-up period of 2 years, and possibly may be less pronounced in the group using the combination of the PI and the NNRTI as a result of the HDL-cholesterol elevating effect of the NNRTI and
4. Mitochondrial DNA depletion relative to nDNA in PBMC’s and adipocytes and adipocyte apoptosis will occurr less in the group using the combination of PI and NNRTI than in the group using the PI and NRTI and
5. Bone mineral density changes may occur in a part of these patients. Ít is not clear whether these changes will differ between the two groups.

**4. Study questions**

The study questions during the first 3 months are:

1. Is there a difference between baseline and follow-up values of the following parameters (see below) in both study groups?
2. Is there a difference in the degree and timing of occurrence of the following parameters between the two study arms?

Parameters:

- insulin resistance
- microvascular function
- blood pressure
- lipid and lipoprotein profile

The study questions during the next 21 months are:

- - - 1. Is there a difference between baseline and follow-up values of the following parameters (see below) in both study groups?
      2. Is there a difference in the degree and timing of occurrence of the following parameters between the two study arms?

Parameters:

- insulin resistance
- microvascular function
- blood pressure
- lipid and lipoprotein profile
- the degree of lipo-atrophy and lipohypertrophy
- arterial vessel wall function/ morphology
- the amount of mitochondrial DNA relative to nDNA in PBMC’s and in peripheral fatty tissue
- the degree of apoptosis in peripheral fatty tissue
- bone mineral density (total body, proximal femur and lumbar spine)

In addition, correlations between these parameters will be made.

**5. Methods**

**5.1 Subjects**

We will study 2 groups of 30 patients each. All patients have HIV-1 infection (positive anti-HIV-1 antibodies determined with ELISA and confirmed with Western blot technique).

In each group the first 8 patients will undergo hyperinsulinemic euglycemic clamp studies. Microvascular measurements will also only be done in these patients. The other studies will be done in all patients.

**5.2 Inclusion criteria**

- age between 18 and 70 years.
- no prior use of antiretroviral therapy
- indication for antiretroviral treatment according to common standards

(current standards: CDC-category B or C (or: symptomatic HIV-infection); or CDC-category A (asymptomatic HIV-infection) and CD4 cells < 350/mm3; CD4 cells 350-500/mm3 and HIV-RNA > 5000 copies/ml; CD4 cells > 500/mm3 and HIV-RNA > 30.000 copies/ml) (38,39)

## **5.3 Exclusion criteria**

- female sex
- Body Mass Index (kg/m^2^) > 35.
- Known history of diabetes mellitus or hyperlipidemia
- History of diabetes mellitus in a first degree relative
- Use of coenzyme A reductase inhibitor or fibric acid derivative in the last 6 weeks before inclusion
- use of the following medication: systemic corticosteroids, thiazide diuretics (influence on triglycerides and glucose/insulin resistance), calcium-entry blockers, angiotensin-converting inhibitors, nitrates (influence on macro/microvascular measurements)
- Use of nandrolon or testosteron
- Any disorder or condition which can be expected to lead to lessened compliance with the study protocol.

**5.4 Trial design**

The trial will be designed as a randomised clinical trial.

The study should be regarded as an exploratory trial aiming to increase our understanding of the pathogenesis of the metabolic changes which may occur as a result of antiretroviral combination therapy.. All analyses will be descriptive in nature, comparing patients both as their own control and between treatment arms.

**5.5 Randomisation and blinding**:

Participants will be randomized with a computer program, using a minimization strategy for Body Mass Index below or above 25. Before being randomized all patients will be asked to undergo the hyperinsulinemic euglycemic clamp until a number of 8 patients in both study arms have been reached. In order to guarantee optimal balance between the two allocations, stratification for participation in the clamp will be done.

The investigators performing the measurements of the study parameters will be blinded to the allocated treatment. The patients and treating physicians will not be blinded.

**5.6 Intervention/ study arms**:

1. Lopinavir/ ritonavir (Kaletra®) 400/100 mg bid +Zidovudine/ lamivudine (Combivir®), 300/150 mg bid, both per os. (PI + NRTI)
2. Lopinavir/ ritonavir (Kaletra®) 533/133 mg bid + nevirapine (Viramune®) 200 mg bid both per os. Starting dose of nevirapine will be 200 mg once daily, after 14 days 200 mg bid. (PI + NNRTI)

An increased dose of Lopinavir/ritonavir is used in the second group to obtain the same plasma concentration of the drug as in the first group. This is based on the induction of the activity of CYP3A by NNRTI, leading to decreased plasma concentrations of Lopinavir/ritonavir (40).

Combination regimens of Lopinavir/ritonavir with NRTI and with NNRTI have both been studied (41,42). Both regimens are effective and well-tolerated treatment strategies for patients with HIV-infection. Although no comparative studies have been done, both treatment strategies are considered to be equally effective.

All study medications used in the study are licensed for use in HIV-infected patients in the Netherlands.

# 5.7 Usual patient care

Patients will be treated by their treating physicians. As in usual patient care, history, general physical examination and laboratory investigations will be done at baseline, 4 weeks after starting treatment and every 3 months.

Laboratory investigations according to standard patient care include:

- lymphocyte subsets (every 3 months)
- plasma HIV-RNAload (every visit)
- hepatitis B- and C- serology (baseline and when appropriate)
- plasma concentration lopinavir/ritonavir and nevirapine (when appropriate)
- hematology (hemoglobin, thrombocyte and leukocyte count, Mean Cell Volume, Mean Cell Hemoglobin, MCHC) (every visit)
- chemistry (sodium, potassium, creatinine, uric acid, ASAT, ALAT, alkaline phosphatase, gamma GT, bilirubin, amylase, CK, glucose, lactate, total cholesterol and triglycerides (non fasting)) (every visit)

**5.8 Description of specific methods**

A number of investigations will be done in addition to usual patient care. All these investigations will be done at baseline and 3, 12 and 24 months after starting treatment. The following will be done:

- - - 1. Laboratory investigations:
- Fasting lipid profile (total cholesterol, HDL-, LDL-cholesterol, apolipoproteins A and – B).
- HbA1C, fasting insulin, glucose.
- Mitochondrial DNA relative to nDNA in PBMC’s (NASBA technique)
- storage plasma and PBMC’s

2. Body composition measurements:

- body mass index (weight/ (length²))
- Waist-to-hip ratio measured by a steel tape at the smallest circumference of the abdomen and the largest circumference of the hips.
- Skinfoldthickness using a skinfold caliper measured at the level of the m. biceps brachii, m. triceps brachii (both halfway the upper arm with the arm in exorotation), subscapular (just below the inferior angle of the scapula) and supra-iliacal (just above the crista iliaca, in the midaxillar line), all on the right side of the body.
- Mid-arm circumference of the left arm in right handed persons and the right arm in left handed persons, measured halfway between acromion and olecranon.
- bio-electrical impedance analysis (uses total body electrical resistance by which lean body mass can be calculated)
- Computer tomography (CT)-scan: quantitative assessment of intra-abdominal fatty tissue. Single slice CT passing through umbilicus (fourth lumbar vertebra) for calculation of visceral, subcutaneous and total abdominal adipose tissue.
- Dual-energy x-ray absorptiometry (DEXA): for quantitative assessment total body water, fat and bone. In addition, at baseline and 24 months, a regional DEXA will be done for assessment of bone mineral density in the proximal femur and lumbar spine.

# 3. Insulin resistance measurements (in a subset of patients):

# To measure insulin resistance we will use the hyperinsulinemic, euglycemic clamp technique as described before in this patientgroup (13). This method will enable us to measure endogenous glucose production, lipolysis and peripheral glucose uptake .

Following a 12 hour fast, a catheter is inserted in the antecubital vein of each arm. One catheter is used for sampling of arterialized blood using a heated handbox (60 °C). The other catheter is used for infusion of [6,6-^2^H_2_]-glucose, glucose 20% and insulin. At 09.00 hrs. (t= - 2 hr.), after drawing a blood sample for background enrichment of plasma glucose and glycerol, a continuous infusion of [6,6-^2^H_2_]glucose is started at a rate of 0.22 μmol·kg^-1^·min^-1^ after a priming dose is administered which equals 80 minutes of infusion. At 10.00 hrs. a primed dose of 1,6 μmol/kg D5-glycerol is given after which a infusion of 0,11 μmol/kg/min D5-glycerol is started and continued untill the end of the study.

After 120, 130, 140 and 150 minutes blood samples are drawn for determination of basal endogenous glucose production and lipolysis. FFA concentrations are measured at 120 and 150 minutes. Subsequently at t= + 0,5 hr. a primed continuous infusion of insulin is started for 2,5 hours at a rate of 20 mU·m^-2^ body surface area·min^-1^.

Plasma glucose concentration is measured every 5 minutes and glucose 20% is infused at a variable rate to maintain plasma glucose at 5.0 mmol/L. [6,6-^2^H_2_] glucose is added to the 20 % glucose solution to achieve glucose enrichments of 2 % to minimize changes in isotopic enrichment due to changes in the infusion rate of exogenous glucose, and thus to allow for accurate quantification of endogenous glucose production.

The last hour of insulin infusion every 10 minutes blood samples are drawn for determination of endogenous glucose production and lipolysis. FFA concentrations are measured at t= 2,5 hr. and at t=3 hr. During the study subjects are only allowed to drink water.

Hyperinsulinemic, euglycemic clamp procedures will be postponed in case of

corticosteroid use or intercurrent disease with fever in the last 2 weeks.

1. Macrovascular function and morphology assessment

All measurements are conducted by one observer, in a temperature-controlled room. Blood pressure and heart rate are recorded on the left upper arm at 5-minutes intervals using an oscillometric device (BP-8800, Colin, Komaki City, Japan). Upper arm blood pressure is used for calibration of the tonometrically obtained pressure curve of the radial artery (Millar type SPT-301 pencil applanation tonometer, Millar Instruments, Houston, USA).

During the measurement, after at least 15 minutes of rest in the supine position, the left carotid artery is checked for the existence of atherosclerotic plaques by B-mode ultrasound. Carotid artery diameter is measured 10 mm proximal to the carotid bifurcation. A video-printout is made indicating the position of the M-line in relation to the bifurcation in order to perform the second measurement at the same cross-section of the artery. Wall tracking of the right carotid artery is performed, diameter and distensibility are recorded. Intima/media thickness assessment of the carotid artery is done with the wall track system (software). The femoral artery is measured 15 mm proximal to the bifurcation of this artery into the deep and superficial femoral artery. Again, a printout is made for re-investigation of the artery later.

Furthermore, radial artery pressure will be determined using applanation tonometry. This is a non-invasive method to determine the pressure wave in the aorta by measuring the pressure curve in the radial artery.

5. Microvascular function assessment (in a subset of patients):

- Capillary recruitability

Nailfold capillaries in the dorsal skin of the third finger will be visualized by a capillary microscope, linked to a television camera, a videorecorder and a monitor. A magnification of 99% is used. Capillary density is defined as the number of erythrocyte-perfused capillaries per square millimeter of nailfold skin. Percentage capillary recruitment is calculated by dividing the increase in capillary density after 4 minutes of arterial occlusion of the third finger by a cuff by baseline capillary density.

- Endothelium dependent vasodilation of the skin microcirculation is evaluated by iontophoresis of acetylcholine and endothelium independent vasodilation with sodium nitroprusside, in combination with laser Doppler fluxmetry in the skin. (Iontophoresis is a non-invasive method of drug application using a small electric current to transfer charged substances across the skin locally.)

1. Adipose tissue biopsy

To measure the amount of mitochondrial DNA relative to nDNA and assess the degree of apoptosis in fatty tissue a biopsy of subcutaneous adipose tissue on the antero-lateral aspect of the right leg or on the buttocks is done.

Apoptosis assessment is done by measurement of caspase 3 activation in adipocytes and indirect assessment of caspase 9 activation through p53 upregulation. Assessment of mitochondrial DNA content relative to nDNA is done by the NASBA technique (nucleic acid sequence-based amplification).

Fatty tissue will be stored for possible future use.

1. Questionnaires

Questionnaires on self report of body shape changes and on quality of life will be administered.

1. Current medication use, smoking, alcohol consumption and intercurrent illnesses including cardiovascular diseases will be recorded. Patients will be asked some questions concerning diet and/or exercise level during the study period to get insight into possible changes in these parameters.

**5.9 Possible side effects during study**

Side effects of many antiretrovirals, including the compounds used in this study, have been described. Action on side effects will be the same as in normal patient care. Some specific actions are mentioned in this section.

When rash occurs in patients using nevirapine, Cetirizine (Zyrtec®) 10 mg once daily will be given. In case of persisting rash nevirapine will be stopped and substituted by Efavirenz (Stocrin®) 600 mg once daily.

Withdrawal because of side effects: see paragraph below.

**Actions to side effects that are specifically studied in this study**

1. Elevated triglycerides and/or cholesterol.

In case of fasting triglycerides above 15 mmol/L gemfibrozil 900 mg once daily will be started.

In case of a fasting total cholesterol above 8.5 mmol/L a cholesterol lowering diet is prescribed. When this high value persists after 3 months, pravastatine 20 mg once daily (maximal dose 40 mg once daily) will be started. The period of three months is chosen according to current general guidelines to observe the effect of a dietary intervention. It will also allow us to to study the initial set of measurements without interference of lipid-lowering therapy, while at the same time not exposing the subjects to the risk of long-term hyperlipidemia.

2. Elevated plasma glucose levels

Therapy will be started if plasma glucose levels above 7 mmol/L in the fasting state or above 11.1 mmol/L in the non-fasting state are found (according to the international definition of diabetes mellitus (43)). Therapy will start with Metformin 500 mg tid as recommended in national guidelines (38).

# 6. Data

## **6.1 Data recording**

Each patient will be assigned a study number. A Case Record File (paper and electronic) will be kept per patient containing all study data. These Case Record Files will not be destroyed until 15 years after inclusion.

# 6.2 Statistical analysis

For comparison between baseline and follow-up parameters a paired t-test will be used.

For comparison between study arms the Students t test or Whitney U test will be used where appropriate.

# 6.3 Data presentation and publishing

The results of this study will be published if applicable. Financial pharmaceutical sponsors will receive manuscripts at least 30 days before publication in order to be able to provide comments, but will have no binding influence on contents of the final manuscripts.

# 7. Ethical considerations

**7.1 General**

The study will be conducted according to the principles of the “Declaration of Helsinki” (as amended in Edinburgh, October 2000).

# 7.2 Recruitment, consent and withdrawal

Subjects will be recruited from outpatient clinics of the above mentioned hospitals. Oral and written explanation about the study will be given by the investigators of the study. Subjects will be given one week time (or more if wished) for considering participation in the study. The subjects will be included in the study after they have given written acknowledgement of informed consent to participate. After approval of the subjects their general practitioners will be notified. Subjects can withdraw from the study at any time for any reason if they wish to do so without any consequences. The responsible investigator can withdraw a subject if continuing participation is in his opinion deleterious for the subjects well-being.

Stopping or changing therapy because of serious side effects due to medications or because of virological failure is done as in normal patient care. In both cases subjects will be withdrawn from the study.

If a patient drops out of the study for whatever reason, all investigations planned for the next visit will be performed at the time of discontinuation with the patients consent.

Patients will be paid travel expenses for every hospital visit which is in addition to regular patient care.

**7.3 Description of burden and possible risk of the study for subjects enrolled in the study**:

Patients will visit the two main investigating hospitals (VUmc and AMC) on four occasions during the 2 years of follow-up. These visits will be in addition to the visits to their treating physicians which are considered as normal patient care.

On each of these four occasions all investigations will be done on two separate days if possible. On the first day, which will take place in the VUmc, body composition measurements (except for CT and DEXA), microvascular and macrovascular measurements are done in addition to fatty tissue biopsy, blood withdrawal and completion of questionnaires. On the second day, which will take place in the AMC, the hyperinsulinemic, euglycemic clamp (in subset of patients) and CT and DEXA will be done. The first day is estimated to take 3 hours for the subset of patients undergoing microvascular measurements and 1.5 hours for the other patients. The second day will take approximately 6 hours for patients undergoing the hyperinsulinemic euglycemic clamp and 1 hour for the other patients.

Of the above mentioned investigations, body composition measurements (except for CT and DEXA), BIA, macrovascular measurements (performed by ultrasound) and capillary recruitment assessment are not considered to be potentially harmful to patients.

The radiation hazard of the DEXA is 0.0026 milliSievert per total body measurement and 0.0052 milliSievert when regional bone density measurements are included. The radiation hazard of one single-slice CT is 0.4 milliSievert . Total radiation exposure in two study years is therefore 1.6156 milliSievert. For comparison: background radiation is 2 millisievert per year for every person.

The amount of blood withdrawn (69 ml on 4 occasions in two years, total 276 ml) is not considered to be of negative influence to the subject’s health. During the clamp procedure an additional 100 ml of blood will be withdrawn. The amount of blood drawn from the subjects undergoing the hyperinsulinemic euglycemic clamp procedure will therefore be 169 ml on 4 occasions (69 ml on first day and 100 ml on second day), total 676 ml in two years.

In the measurement of endothelium (in) dependent vasodilation iontophoresis is used in combination with Laser Doppler. Iontophoresis of acetylcholine and nitroprusside causes only local effects on skin perfusion and is considered safe. Products containing lasers are classified according to the laser output and the radiation hazards to individuals exposed to the radiation. The classes range from 1 (lowest risk) to 4. The used laser Doppler system for this study is a class 1 laser product.

The hyperinsulinemic, euglycemic clamp procedure may be considered a burden. No negative effects (immediate or long-term) on the health of the subjects have been reported. Glucose levels are measured frequently (at least every five minutes) and adjusted by infusion of glucose. Medical personnel is present at all times.

In our experience, fatty tissue biopsy is tolerated well in HIV- infected patients and leaves minimal scarring.

**7.4 Subject privacy and data handling**

All study data will be handled confidentially.

**7.5 Independent physician**

Dr. J. Schouten, internist, VUMC, department of internal medicine, telephone 020-4444444, *185, will act as an independent physician for this study.

# 7.6 Unexpected findings

Unexpected findings with medical consequences of study investigations will be communicated with the patient.

# 7.7 Compensation for injury

The insurance meets the legal obligations set by the “Dutch Medical Research Involving Human Subjects Act” (dated February 26,1998).

**8. Study duration**:

The study duration will be 24 months.

**9. References**

1. Carr A. An objective case definition of HIV lipodytrophy. 9^th^ Conference on retroviruses and opportunistic infections, Seattle, 24-28 February 2002, Abstract 31.
2. Survival after introduction of HAART in people with known duration of HIV-1 infection. Lancet 2000, 355:1158-9.
3. Palella FJ Jr, Delaney KM, Moorman AC, Loveless MO, Fuhrer J, Satten GA, Aschman DJ, Holmberg SD. Declining morbidity and mortality among patients with advanced human immunodeficiency virus infection. N Engl J Med 1998, 338:853-60.
4. Carr A, Samaras K, Burton S, Law M, Freund J, Chishol DJ Cooper DA. A syndrome of peripheral lipodystrophy, hyperlipidemia and insulin resistance in patients receiving HIV protease inhibitors. AIDS 1998,12:F51-58.
5. Carr A, Samaras K, Thorisdottir A , Kaufmann GR, Chisholm DJ, Cooper DA. Diagnosis, prediction, and natural course of HIV-1 protease-inhibitor-associated lipodystrophy, hyperlipidemia, and diabetes mellitus: a cohort study. Lancet 1999, 353:2093-99.
6. Martinez E, Mocroft A, Garcia-Viejo M, Pérez-Cuevas JB, Blanco JL, Mallolas J, Bianchi L, Conget I, Blanch J, Gatell JM . Risk of lipodystrophy in HIV-1 infected patients treated with protease inhibitors: a prospective cohort study. Lancet 2001,357:592-98.
7. Kohrt WM. Preliminary evidence that DEXA provides an accurate assessment of body composition. J Appl Physiol 1998;84:372-77.
8. Schoen RE, Thaete FL, Sankey SS, Weissfeld JL, Kuller LH. Sagittal diameter in comparison with single slice CT as a predictor of total visceral adipose tissue volume. Int J Obes Relat Metab Disord 1998;22:338-42.
9. Schwenk A, Breuer P, Kremer G, Ward L. Clinical assessment of HIV-associated lipodystrophy syndrome: bio-electrical impedance analysis, anthropometry and clinical scores. Clin Nutr 2001;20:243-9.
10. Brambilla P, Bricalli D, Sala N, Renzetti F, Manzoni P, Vanzulli A, Chiumello G, di Natale B, Vigano A. Highly active antiretroviral-treated HIV-infected children show fat distribution changes even in absence of lipodystrophy. AIDS 2001;15:2415-22.
11. Ferrannini E, Mari A. How to measure insulin sensitivity. J Hypertens 1998;16:895-906.
12. Noor MA, Lo JC, Mulligan K, Schwarz J, Halvoresen RA, Schambelan M, Grunfeld C. Metabolic effects of indinavir in healthy HIV-seronegative men. AIDS 2001;15:11-18.
13. Van der Valk M, Bisschop PH, Romijn JA,Ackermans MT, Lange JMA, Endert E, Reiss P, Sauerwein HP. Lipodystrophy in HIV-1-positive patients is associated with insulin resistance in multiple metabolic pathways. AIDS 2001;15:2093-2100.
14. Behrens G, Dejam A, Schmidt H, Balks H, Brabant G, Körner T, Stoll M, Schmidt RE. Impaired glucose tolerance, beta cell function and lipid metabolism in HIV-patients under treatment with protease inhibitors. AIDS 1999;13:F63-70.
15. Van der Valk M, Kastelein JJP, Murphy RL, van Leth F, Katlama C, Horban A, Glesby M, Behrens G, Clotet B, Stellato RK, Molhuizen HOF, Reiss P. Nevirapine-containing antiretroviral therapy in HIV-1 infected patients results in an anti-atherogenic lipid profile. AIDS 2001;15:2407-2414.
16. Sattler FR, Qian D, Louie S. Elevated blood pressure in subjects with lipodystrophy. AIDS 2001;15:2001-10.
17. Tebas P, Powderly WG, Claxton S, Marin D, Tantisiriwat W, Teitelbaum SL, Yarasheki KE. Accelerated bone mineral loss in HIV-infected patients receiving potent antiretroviral therapy. AIDS 2000;14:F63-7.
18. Carr A, Miller J, Eisman JA, Cooper DA. Osteopenia in HIV-infected men: association with asymptomatic lactic acidemia and lower weight pre-antiretroviral therapy. AIDS 2001;15:703-9.
19. Carr A, Samara K, Chisholm DJ, Cooper DA. Pathogenesis of HIV-1-protease inhibitor-associated peripheral lipodystrophy, hyperlipidemia, and insulin resistance. Lancet 1998;351:1881-83.
20. Brinkman K, Smeitink JA, Romijn JA, Reiss P. Mitochondrial toxicity induced by nucleoside-analogue reverse transcriptase inhibitors is a key factor in the pathogenesis of antiretroviral-therapy-related lipodystrophy. Lancet 1999;354:1112-15.
21. Van Gemen B, de Ronde A, Casula M, Timmermans E, Van Dooren M, Westrop M, Dobbelaar I, Bakker M, Weverling GJ, Lange J, Goudsmit J, Reiss P. Quantification of mitochondrial DNA relative to nuclear DNA in peripheral blood mononuclear cells: validation of an accurate duplex assay for use in patients undergoing antiretroviral therapy. Antiviral therapy 2001;6 (supplement 4):14.
22. Côté HCF, Brumme ZL, Craib KJP, Alexander CS, Wynhoven B, Ting L, Wong H, Harris M, Harrigan PR, O’Shaughessy MV, Montaner JSG. Changes in mitochondrial DNA as a marker of nucleoside toxicity in HIV-infected patients. N Engl J Med 2002;346:811-20.
23. McComsey G, Tan D-J, Lederman M, Wilson E, Wong L-J. Analysis of the mitochondrial DNA genome in the peripheral blood leukocytes of HIV-infected patients with or without lipatrophy. AIDS 2002;16:513-8.
24. Shikuma CM, Hu N, Milne C, Yost F, Waslien C, Shimizu S, Shiramizu B. Mitochondrial DNA decrease in subcutaneous adipose tissue of HIV-infected individuals with peripheral lipoatrophy. AIDS 2001;15:1801-09.
25. Domingo P, Matias-Guiu X, Pujol RM, Francia E, Lagarda E, Sambeat MA, Vázquez G. Subcutaneous adipocyte apoptosis in HIV-1 protease inhibitor-associated lipodystrophy. AIDS 1999;13:2261-67.
26. Van der Valk M, Gisolf EH, Reiss P, Wit FW, Japour A, Weverling GJ, Danner SA. Increased risk of lipodystrophy when nucleoside analogue reverse transcriptase inhibitors are included in the treatment of HIV-1 infection. AIDS 2001,15:847-55.
27. Mallal SA, John M, Moore CB, James IR, McKinnon EJ. Contribution of nucleoside analogue reverse transcriptase inhibitors to subcutaneous fat wasting in patients with HIV infection. AIDS 2000;14:1309-16.
28. Martinez E, Conget I, Lozano L, Casamitjana R, Gatell JM. Reversion of metabolic abnormalities after switching from HIV-1 protease inhibitors to nevirapine. AIDS 1999;13:805-10.
29. Barreiro P, Soriano V, Blanco F, Casimiro C, de la Cruz JJ, González-Lahoz J. Risks and benefits of replacing protease inhibitors by nevirapine in HIV-infected subjects under long-term successful triple combination therapy. AIDS 2000;14:807-812.
30. Kaplan NM. The deadly quartet. Upper body obesity, glucose intolerance, hypertriglyceridemia, and hypertension. Arch Intern Med 1989;149:1514-20.
31. Henry K, Melroe H, Huebsch J. Severe premature coronary artery disease with protease inhibitors. Lancet 1998;351:1328.
32. Grobbee DE, Bots ML. Carotid artery intima-media thickness as an indicator of generalized atherosclerosis. J Int Med. 1994;236:567-73.
33. O'Leary DH, Polak JF, Kronmal RA, Manolio TA, Burke GL, Wolfson SK Jr. Carotid-artery intima and media thickness as a risk factor for myocardial infarction and stroke in older adults: Cardiovascular Health Study Collaborative Research Group. N Engl J Med 1999;340:14-22.
34. Maggi P, Serio G, Epifani G, Fiorentino G, Saracino A, Fico C, Perilli F, Lillo A, Ferraro S, Gargiulo M, Chirianni A, Angarano G, Regina G, Pastore G. Premature lesions of the carotid vessels in HIV-1-infected patients treated with protease inhibitors. AIDS 2000;14:123-128.
35. Serne EH, Stehouwer CD, ter Maaten JC, ter Wee PM, Rauwerda JA, Donker AJ, Gans RO. Microvascular function relates to insulin sensitivity and blood pressure in normal subjects. Circulation 1999;99:896-902.
36. Muurahainen N, Glesby M,Falutz J, Kleintop M, Balser J, Pettit R. Different factors are associated with lipohypertrophy and lipoatrophy in HIV patients with fat maldistribution. AIDS 2000;14 (Suppl 4):S59-60.
37. Pernerstorfer-Schoen H, Jilma B, Perschler A, Wichlas S, Schindler K, Schindl A, Rieger A, Wagner OF, Quehenberger P. Sex differences in HAART-associated dyslipidaemia. AIDS 2001;15:725-34.
38. CBO-richtlijn Antiretrovirale behandeling in Nederland december 2000.
39. Carpenter CCJ, Cooper DA, Fischl MA et al. Antiretroviral Therapy in Adults. Updated Recommendations of the International AIDS-Society -USA Panel. JAMA 2000;283:381-90.
40. Bertz R, Lam W, Hsu A, Rynkiewicz K, Foit C, Bryan P, Sylte J, Brun S, Granneman GR, Sun E. Assessment of the Pharmacokinetic interaction between ABT-378/ritonavir (ABT-378/r) and Efavirenz (EFV) in healthy volunteers and in HIV+subjects. 40^th^ Interscience Conference on Antimicrobial Agents and Chemotherapy, Toronto, 2000, abstract 424.
41. Murphy RL, Brun S, Hicks C, Eron JJ, Gulick R, King M, Clinton White A, Benson C, Thompson M, Kessler HA, Hammer S, Bertz R, Hsu A, Japour A, Sun E. ABT-378/ritonavir plus stavudine and lamivudine for the treatment of atiretroviral-naïve adults with HIV-1 infection: 48-week results. AIDS 2001;15:1-9.
42. Danner S, Richards B, King M, Clumeck N, Lazzarin A, Girard PM, Rockstroh J, Becker S, Pantaleo G, Bergmann F, Ho D, Tubiana R, Carosi G, Bertz R, Hsu A, Sylte J, Isaacson J, Kempf D, Sun E. Kaletra (lopinavir/ritonavir) and Efavirenz: 72-week safety and efficacy evaluation in multiple PI-experienced patients. 41^st^ Interscience Conference on Antimicrobial Agents an Chemotherapy, December 2001, poster 1925.
43. Report of the Expert Committee on the diagnosis and classification of diabetes mellitus. Diabetes Care 1997 20(7), 1183-97.
