# Supplementary material for: Zidovudine/Lamivudine for HIV-1 Infection Contributes to Limb Fat Loss
Source: PLoS One. 2009 May 21;4(5):e5647. doi: 10.1371/journal.pone.0005647 (PMC2682584; doi:10.1371/journal.pone.0005647)
Supplement: Protocol Amendment S1 — MEDICLAS study protocol (0.02 MB DOC) [file pone.0005647.s003.doc]

**MEDICLAS** (**M**etabolic **E**ffects of **Di**fferent **Cl**asses of **A**ntiretroviral**S**)

Amendments to the protocol dated 21-03-2002

Date: 27-01-2003

1. Additional participating centers:

An additional participating center will be added: the Royal Free Hospital in London (Investigator: Dr M. Youle). Arrangements have been made for the patients to visit Amsterdam for the study investigations. The patient information has been translated in English and corrected according to the requirements of the Ethics Committee of the Royal Free Hospital. The Ethics Committee of the Royal Free Hospital has given its approval for the study.

We are also talking with the Aurora Hospital in Hus, Finland (Investigator: Dr M. Ristola) to see if participation is possible. The same arrangements will be made as for the patients from London.

2. Additional study investigations:

1. Blood levels of Lopinavir/ritonavir and nevirapine will be done at visits 3, 12 and 24 months. This is currently seen as part of usual patient care.
2. DEXA scans for bone mineral density will be done at every visit (baseline, 3, 12 and 24 months). This was incorrectly stated in the protocol dated 21-03-2002. The patient information letter already included this information and the radiation hazard statement (adviescommissie stralenbelasting, dr H.W. Venema, date 17-04-2002) also already included 4 DEXA scans for bone mineral density.
3. Laboratory investigations done during the study visits will also include sodium, potassium, chloride and bicarbonate, in order to be able to calculate the aniongap. This has become one of the parameters used in the case definition of lipodystrophy. No additional blood withdrawal is necessary.
4. Computer tomograpy: during the single slice CT scan used to quantify visceral adipose tissue, we will also measure lumbar spine bone mineral density using quantitative computed tomography. This is done from the same single slice image, and there is no additional time or radiation hazard involved. Quantitative computed tomography measures trabecular bone, whereas DEXA measures primarily cortical bone. Trabecular bone may be lost at a greater rate than cortical bone in patients with HIV lipodystrophy (1). Using both techniques gives us the opportunity to study bone loss more extensively.
5. Macrovascular function and morphology assessment: Additionally, diameter and distensibility of the brachial artery will be measured by ultrasound. This is used to determine the pressure wave in the aorta in a non-invasive way. Recently, this method was described as complementary and possibly superior to the applanation tonometry method (2). For this study, both methods will be used. For this investigation, the subject has to lie in the supine position for an additional five minutes. No radiation hazard is involved.

3. Withdrawal:

Subjects will not be withdrawn from the study if therapy is stopped or changed, unless at their own request. An intention to treat analysis will be done.

Literature:

1. Huang JS, Rietschel P, Hadigan CM et al. Increased abdominal visceral fat is associated with reduced bone density in HIV-infected men with lipodystrophy. AIDS 2001,15:975-982.
2. Van Bortel LM, Balkestein EJ, Van der Heijden-Spek JJ et al. Non-invasive assessment of local arterial pulse pressure: comparison of applanation tonometry and echo-tracking. J Hypert 2001;19: 1037-44.
