# Supplementary material for: Zidovudine/Lamivudine for HIV-1 Infection Contributes to Limb Fat Loss
Source: PLoS One. 2009 May 21;4(5):e5647. doi: 10.1371/journal.pone.0005647 (PMC2682584; doi:10.1371/journal.pone.0005647)
Supplement: Protocol Amendment S2 — MEDICLAS study protocol (0.03 MB DOC) [file pone.0005647.s004.doc]

**MEDICLAS** (**M**etabolic **E**ffects of **Di**fferent **Cl**asses of **A**ntiretroviral**S**)

(protocol 02-72)

Amendment 2 to the protocol dated 21-03-2002

Date: October 20, 2003

# Introduction

Recently, promising new data on gene expression in subcutaneous adipose tissue were published, providing new insight into the mechanisms underlying the loss of subcutaneous fat in highly active antiretroviral therapy (HAART) related lipodystrophy. Messenger RNA concentrations of certain adipocyte transcription factors were found to be significantly lower in HIV-positive patients with lipodystrophy than in those without lipodystrophy. Through inhibition of adipocyte differentiation, these changes could contribute to the loss of subcutaneous fat in patients with HAART-associated lipodystrophy. Gene expression of proteins in fatty acid metabolism and in glucose transport was similarly decreased, which may point towards the underlying defects for the metabolic disturbances of the lipodystrophy syndrome (1).

This cross-sectional study however does not clarify the sequence of events leading to these alterations in adipose tissue gene expression. The role of the underlying HIV infection itself is not clear, and neither is the contribution of the different classes of antiretroviral drugs. In vitro studies show a possible effect on adipose tissue differentiation by two antiretroviral drug classes, particularly of the protease inhibitors (PI) and to a less clear extent of the nucleoside reverse transcriptase inhibitors (NRTI) (2-7).

# Objective for amendment

The MEDICLAS study offers a unique possibility to study the time course of changes in gene expression in adipose tissue, because it is a prospective study in which patients are studied both before and after starting antiretroviral therapy. Furthermore, one treatment arm is NRTI sparing, providing to some extent the opportunity to study the effect of the different drug classes separately.

In the MEDICLAS study, an adipose tissue percutaneous punch biopsy is already done from the upper leg for determination of mitochondrial DNA content and apoptosis. However, the amount of fatty tissue collected from this procedure is not enough for determination of gene expression profiles. For this purpose, a fatty tissue aspiration should be done from the abdominal subcutaneous fat, in order to obtain a larger amount of adipose tissue.

# Technique of the fatty tissue aspiration

After local anesthesia, a needle is inserted into a fold of the abdominal subcutaneous fat, and fatty tissue is aspirated by repeated movements. This technique has been applied repeatedly by J. Sutinen, the Finnish co-investigator for the MEDICLAS study, in 80-90 patients, without serious complications. One patient was seen with bleeding after the aspiration, which was due to not compressing the puncture site adequately. In all of the following patients, the aspiration site was compressed for 15 minutes, and no bleeding was seen. No cases of infection have been seen. One patient complained of pain after the aspiration, which was adequately treated with a simple analgesic drug (paracetamol). No patient refused a second aspiration. Although there is bruising (small hematomas up to 2 cm in diameter) in about 80% of patients, scars are almost never seen.

# Conclusion

This additional procedure is a safe technique, causing minimal discomfort to the patient, and offering the possibility of improving our understanding of the underlying molecular defects of HAART related lipodystrophy. For that reason, we want to include this investigation in the MEDICLAS study. It will be done in addition to the fatty tissue punch biopsy in the upper leg, as mitochondrial DNA quantification cannot be performed in fat collected by aspiration and it is not known whether apoptosis can be assessed reliably on such material.

References:

1. Kannisto K, Sutinen J et al. Expression of adipogenic transcrition factors, peroxisome proliferator-activated receptor gamma co-activator 1, IL-6 and CD45 in subcutaneous adipose tissue in lipodystrophy associated with highly active antiretroviral therapy. AIDS 2003;17(12):1753-1762.
2. Caron M, Auclair M, Vigouroux C, Glorian M, Forest C, Capeau J. The HIV protease inhibitor indinavir impairs sterol regulatory element-binding protein-1 intranuclear localization, inhibits preadipocyte differentiation, and induces insulin resistance. *Diabetes* 2001; 50:1378–1388.
3. Dowell P, Flexner C, Kwiterovich PO, Lane MD. Suppression of preadipocyte differentiation and promotion of adipocyte death by HIV protease inhibitors. *J Biol Chem* 2000; 275:41325–41332.
4. Lenhard JM, Furfine ES, Jain RG, Ittoop O, Orband-Miller LA, Blanchard SG, *et al*. HIV protease inhibitors block adipogenesis and increase lipolysis *in vitro*. *Antiviral Res* 2000; 47:121–129.
5. Wentworth JM, Burris TP, Chatterjee VK. HIV protease inhibitors block human preadipocyte differentiation, but not via the PPARgamma/RXR heterodimer. *J Endocrinol* 2000; 164:R7–R10.
6. Zhang B, MacNaul K, Szalkowski D, Li Z, Berger J, Moller DE. Inhibition of adipocyte differentiation by HIV protease inhibitors. *J Clin Endocrinol Metab* 1999; 84:4274–4277.
7. Roche R, Poizot-Martin I, Yazidi CM, Compe E, Gastaut JA, Torresani J, *et al*. Effects of antiretroviral drug combinations on the differentiation of adipocytes. *AIDS* 2002; 16:13–20.
