# Supplementary material for: Zidovudine/Lamivudine for HIV-1 Infection Contributes to Limb Fat Loss
Source: PLoS One. 2009 May 21;4(5):e5647. doi: 10.1371/journal.pone.0005647 (PMC2682584; doi:10.1371/journal.pone.0005647)
Supplement: Protocol Amendment S3 — MEDICLAS study protocol (0.03 MB DOC) [file pone.0005647.s005.doc]

**MEDICLAS** (**M**etabolic **E**ffects of **Di**fferent **Cl**asses of **A**ntiretroviral**S**)

(protocol 02-72)

Amendment 3 to the protocol dated 21-03-2002

Date: February 1, 2005

Since MEDICLAS began, it is becoming increasingly clear that longer term comparative data between different treatment strategies on lipodystrophy and metabolic complications are scarce but extremely important for improving our understanding of what may be less harmful treatments. Published prospective studies on metabolic changes rarely have a follow-up of longer than 1-2 years. The only small study with a longer follow-up suggests that objective changes in body composition correlating with HIV associated lipodystrophy only become apparent after two years of antiretroviral therapy (1). This was a non-randomized study using various antiretroviral regimens, which did not measure metabolic changes in the in-depth way the MEDICLAS study does.

We therefore propose to extend the follow up of MEDICLAS to three years after start of antiretroviral therapy. In practice the proposed study amendment would imply the addition of an additional comprehensive study visit for each patient at three years. This study visit will include laboratory investigations, body composition measurements (body mass index, waist-to-hip ratio, skinfoldthickness, bio-electrical impedance analysis, computer tomography, dual-energy x-ray absorptiometry (DEXA)), macrovascular function and morphology assessment, adipose tissue biopsies and questionnaires as described in the original protocol.

For the subset of patients who participate in the substudy with euglycemic hyperinsulinemic clamps, we would also be adding such a clamp and microvascular function assessment (capillary recruitability and iontophoresis of acetylcholine and sodium nitroprusside) at the three year timepoint.

Patients will continue to be treated by their treating physicians. As in usual patient care, history, general physical examination and laboratory investigations will continue to be done every 3 months.

A new patient information letter will be given to all participants. If they agree to continue the study, a new informed consent should be signed.

Reference

1. Mallon P, Miller J, Cooper D et al: Prospective evaluation of the effects of antiretroviral therapy on body composition in HIV-1 infected men starting therapy. AIDS 2003; 971-979.
