# Supplementary material for: Zidovudine/Lamivudine for HIV-1 Infection Contributes to Limb Fat Loss
Source: PLoS One. 2009 May 21;4(5):e5647. doi: 10.1371/journal.pone.0005647 (PMC2682584; doi:10.1371/journal.pone.0005647)
Supplement: Protocol Amendment S4 — MEDICLAS study protocol (0.03 MB DOC) [file pone.0005647.s006.doc]

**MEDICLAS** (**M**etabolic **E**ffects of **Di**fferent **Cl**asses of **A**ntiretroviral**S**)

(protocol 02-72)

Amendment 4 to the protocol dated 21-03-2002

Date: March 22, 2005

Boehringer Ingelheim has issued new guidance concerning initiation of the antiretroviral drug Nevirapine. Last year it was advised that women with HIV infection with CD4 counts greater than 250 cells/mm³ at the time of initiating treatment with Nevirapine have a higher risk of developing severe, life-threatening and potentially fatal hepatotoxicity. This guidance has now been revised to also cover men with CD4 counts greater than 400 cells/mm³.

As only men are enrolled into the MEDICLAS study, a CD4 cell count greater than 400 cells/mm³ will be introduced as an exclusion criterium.

It should be noted that in almost all study sites, treatment for HIV infection is generally initiated at lower CD4 count levels (200-350).
