# Supplementary material for: Zidovudine/Lamivudine for HIV-1 Infection Contributes to Limb Fat Loss
Source: PLoS One. 2009 May 21;4(5):e5647. doi: 10.1371/journal.pone.0005647 (PMC2682584; doi:10.1371/journal.pone.0005647)
Supplement: Protocol Amendment S5 — MEDICLAS study protocol (0.03 MB DOC) [file pone.0005647.s007.doc]

**MEDICLAS** (**M**etabolic **E**ffects of **Di**fferent **Cl**asses of **A**ntiretroviral**S**)

(protocol 02-72)

Amendment 5 to the protocol dated 21-03-2002

Date: December 12, 2005

In the Mediclas study, CT scans are done to quantify visceral and subcutaneous abdominal adipose tissue, and also to measure lumbar spine bone mineral density using quantitative computed tomography (Q-CT). Because measurement of bone mineral density using Q-CT has only been validated at the levels of the 2^nd^ and 3^rd^ lumbar vertebra (L2 and L3), the department of radiology AMC has changed the original procedure (in which only a single slice CT would be done at the level of the 4^th^ lumbar vertebra (L4) for quantifying adipose tissue) to include 2 CT slices (level L2 and L3). All CT scans done for Mediclas up to this date in the AMC (for 38 participants) have thus been done at these 2 levels. For the 12 trial participants enrolled in Barcelona however, CT scans have been locally done at level L4.

Since the Mediclas study started, a case definition for HIV Lipodystrophy has been published resulting from an international study(1). This was a consecutive case-control study in HIV-infected, adult outpatients free of active AIDS recruited at 32 sites globally (including the AMC). Data were collected and potential models generated from 504 randomly selected, clinically identified cases and controls. Models were validated in the remaining 284 subjects. In this internationally accepted case definition of HIV lipodystrophy, a single slice CT of the abdomen at level L4 is used.

Due to the fact that scans on participants in Barcelona versus elsewhere have been performed at different levels, we will be facing a problem when reporting the CT results from Mediclas (given the difference in technique used for Spanish and other participants) and when comparing these with other studies. As body composition is one of the important outcome measures of the study, it is important to try and obtain a better understanding of the potential implications.

We therefore propose to include an extra CT slice at level L4 in all CT scans that still need to be performed in the AMC from now on until the end of the study. In this way we can compare the amount of visceral and subcutaneous adipose tissue between levels L2, L3 and L4 within each participant, while it also allows us to compare results between participants and with other studies. We will have to continue measuring at levels L2 and L3 to be able to follow participants longitudinally and to measure spine bone mineral density.

The number of scans that still need to be done from now on until the end of the study are as follows:

For 16 participants only one scan still has to be done (for the 36 month visit, provided they give informed consent for the study prolongation).

For 15 participants 2 scans still have to be done (24 and 36 months, the second if informed consent for the study prolongation is given).

For 2 participants 3 scans still have to be done (12, 24 and 36 months, the last again only if informed consent for the study prolongation is given).

An estimate of the extra radiation dose is included.

The remaining participants have either dropped out of the study (n=5) or undergo scans in Barcelona (n=12), where the protocol will not be changed.

Reference List

(1) Carr A, Emery S, Law M, Puls R, Lundgren JD, Powderly WG. An objective case definition of lipodystrophy in HIV-infected adults: a case-control study. Lancet 2003; 361(9359):726-735.
